# CRISPR‐Cas9‐mediated mutagenesis of kiwifruit *BFT* genes results in an evergrowing but not early flowering phenotype

**DOI:** 10.1111/pbi.13888

**Published:** 2022-07-26

**Authors:** Dinum Herath, Charlotte Voogd, Matthew Mayo‐Smith, Bo Yang, Andrew C. Allan, Joanna Putterill, Erika Varkonyi‐Gasic

**Affiliations:** ^1^ The New Zealand Institute for Plant and Food Research Limited (Plant & Food Research) Mt Albert Auckland New Zealand; ^2^ School of Biological Sciences University of Auckland Auckland New Zealand

**Keywords:** *Actinidia chinensis*, kiwifruit, CRISPR‐Cas9, *BFT*, dormancy, flowering

## Abstract

Phosphatidylethanolamine‐binding protein (PEBP) genes regulate flowering and architecture in many plant species. Here, we study kiwifruit (*Actinidia chinensis*, *Ac*) PEBP genes with homology to *BROTHER OF FT AND TFL1* (*BFT*). CRISPR‐Cas9 was used to target *AcBFT* genes in wild‐type and fast‐flowering kiwifruit backgrounds. The editing construct was designed to preferentially target *AcBFT2*, whose expression is elevated in dormant buds. *Acbft* lines displayed an evergrowing phenotype and increased branching, while control plants established winter dormancy. The evergrowing phenotype, encompassing delayed budset and advanced budbreak after defoliation, was identified in multiple independent lines with edits in both alleles of *AcBFT2*. RNA‐seq analyses conducted using buds from gene‐edited and control lines indicated that *Acbft* evergrowing plants had a transcriptome similar to that of actively growing wild‐type plants, rather than dormant controls. Mutations in both alleles of *AcBFT2* did not promote flowering in wild‐type or affect flowering time, morphology and fertility in fast‐flowering transgenic kiwifruit. In summary, editing of *AcBFT2* has the potential to reduce plant dormancy with no adverse effect on flowering, giving rise to cultivars better suited for a changing climate.

## Introduction

Abundant and synchronous budbreak and flowering in spring are critical for the productivity of many important temperate fruit crops including the woody perennial vine, *Actinidia chinensis* (kiwifruit). Temperate trees and vines typically undergo short day‐induced growth cessation and leaf drop in autumn. This is followed by dormancy, a bud‐protective period during winter where no visible growth occurs until a prolonged exposure to cold reactivates the ability to grow. These processes are accompanied by major metabolic, physiological and molecular changes (reviewed in Beauvieux *et al*., 2018; Brunner *et al*., 2017; Ding and Nilsson, 2016; Liu and Sherif, 2019; Maurya and Bhalerao, 2017; Singh *et al*., 2017; Yang *et al*., 2021). Having a sufficient period of winter chilling to break dormancy is a key environmental determinant of the degree of budbreak, flowering and fruit set in spring. Thus, temperate fruit productivity may be impeded in growing areas with warm winters and by ongoing global warming, owing to insufficient winter chilling (Atkinson *et al*., 2013; Luedeling, 2012).

Kiwifruit growers have identified warmer temperatures during the winter period as their most pressing climatic risk (Cradock‐Henry, 2017), which is currently overcome by application of undesirable dormancy‐breaking chemicals (Ziosi *et al*., 2015). One of the solutions is the development, selection and breeding of cultivars with improved performance, including low‐chill‐requiring varieties. Thus, our research has focused on investigating the molecular‐genetic regulation of kiwifruit phenology with a particular focus on seasonally controlled cycles of growth and dormancy. Research in this global crop is supported by powerful genomic tools and the ability to stably transform kiwifruit, combined with mutagenesis using CRISPR‐Cas9 gene editing (Brian *et al*., 2021; Pilkington *et al*., 2018; Varkonyi‐Gasic *et al*., 2019). Using transgenic overexpression and more recently gene editing, we were able to show that kiwifruit homologues of MADS‐box genes *SHORT VEGETATIVE PHASE* (*SVP*), a *SUPPRESSOR OF OVEREXPRESSION OF CONSTANS1* (*SOC1*) gene *AcSOC1i* and a *FLOWERING LOCUS C‐LIKE (AcFLCL)* all have roles in different aspects of kiwifruit seasonal phenology (Voogd *et al*., 2015, 2021; Wu *et al*., 2012, 2017, 2018, 2019).

Members of the phosphatidylethanolamine‐binding proteins (PEBP) gene family are present in all eukaryotes and are central to plant development and physiology. The PEBP proteins include homologues of the well‐described *Arabidopsis thaliana* (Arabidopsis) *FLOWERING LOCUS T* (*FT*), encoding FT that functions as a florigen and *TERMINAL FLOWER1* (*TFL1*) and *CENTRORADIALIS* (*CEN*), acting to repress flowering and maintain meristem indeterminacy (reviewed in Jin *et al*., 2021; Périlleux *et al*., 2019; Putterill and Varkonyi‐Gasic, 2016). Previously, we have reported key roles for kiwifruit *FT* and *CEN* genes in controlling reproductive maturity and determinacy (Moss *et al*., 2018; Varkonyi‐Gasic *et al*., 2013, 2019; Voogd *et al*., 2017). These are members of the 13 gene kiwifruit PEBP gene family, of which three are *FT* genes (*AcFT*, *AcFT1* and *AcFT2*). Overexpression of any of the kiwifruit *FT* genes resulted in extremely early flowering of the regenerating tissue *in vitro*, indicating that all have the biochemical ability to promote flowering. Of the three, *AcFT* transcript abundance positively correlates with winter chilling and expression in spring, indicating a likely link of *AcFT* with budbreak and flowering. Two *CEN* genes, *AcCEN* and *AcCEN4,* were the most highly expressed of the five *CEN* genes in axillary buds during active growth. Mutation of these two genes by CRISPR‐Cas9 gene editing led to very early reproductive maturation (Varkonyi‐Gasic *et al*., 2019), increased determinacy and continuous flowering in kiwifruit, indicating that these *CEN* genes probably promote vegetative development and indeterminacy and repress flowering, as observed in other plants. Compact, rapid‐cycling plants produced by CRISPR‐Cas9 gene editing of one or both of these genes has allowed generation of model kiwifruit plants, useful for the analysis of gene function (Akagi *et al*., 2019; Varkonyi‐Gasic *et al*., 2021).

The kiwifruit PEBP family also contains three *BROTHER OF FT AND TFL1* (*BFT)* genes whose predicted proteins form a separate subclade within the CEN/TFL1 lineage (Voogd *et al*., 2017). However, the specific functional roles of *BFT* genes are much less well understood in kiwifruit and in other plants. In Arabidopsis, *BFT* shows a similar floral repressive activity to *TFL1/CEN*‐like genes when constitutively expressed, delaying terminal flower formation and repressing axillary inflorescence development when overexpressed, but without the ability to complement the terminal flower phenotype of *tfl1* mutants (Chung *et al*., 2010; Yoo *et al*., 2010). The absence of BFT did not affect flowering time; however, increased numbers of secondary inflorescences when *bft* mutation was combined with *tfl1* suggested a role in inflorescence meristem development (Yoo *et al*., 2010). Furthermore, a triple *tfl1 atc bft* mutant showed the latest flowering amongst a comprehensive set of mutants of the PEBP gene family in Arabidopsis (Kim *et al*., 2013). *BFT* was induced by stress while flowering of the *bft* mutant was insensitive to stress such as high salinity, suggesting that *BFT* delayed flowering and axillary inflorescence development under stress conditions (Chung *et al*., 2010; Ryu *et al*., 2011, 2014) by competing with FT for binding to the FD transcription factor (Ryu *et al*., 2014). *BFT* homologues in woody perennial plants have also been associated, via their expression patterns, with repression of axillary bud or shoot growth (Carmona *et al*., 2007; Foster *et al*., 2013; Voogd *et al*., 2017), suggesting a conserved role in regulation of axillary meristem activity.

In kiwifruit, amongst the three *AcBFT* genes, qRT‐PCR analysis indicated that *AcBFT2* had a striking expression pattern with elevated transcript accumulation during winter in an axillary bud time‐course, peaking at the time of leaf drop (Voogd *et al*., 2017). This pattern of expression suggested that it may represent a candidate for altering kiwifruit dormancy and chilling requirement. Overexpression of the *AcBFT* genes, particularly *AcBFT2,* in Arabidopsis led to delayed flowering and reduced determinacy, suggesting a role in regulation of flowering (Voogd *et al*., 2017).

Here, we present the results of CRISPR‐Cas9 gene editing of *BFT* genes in kiwifruit. These experiments produced plants displaying an evergrowing phenotype because of delayed onset of growth cessation and leaf drop and earlier budbreak compared with the controls. The plants also produced more branches. These phenotypes were associated with bi‐allelic mutations in *AcBFT2*. Major changes to the global transcriptome of axillary buds at both the onset of cessation of growth in autumn and at the time of budbreak in the *Acbft* mutants were observed compared with control buds. However, unlike mutations in the related *AcCEN* genes, reproductive maturity was not affected in the transgenic plants because they did not flower over 3 years and the plants were not more compact than controls. Furthermore, generating a double *Acbft cen4* mutant by gene editing in the rapid flowering *Accen4* mutant background indicated that the *Acbft* mutation did not appear to interfere with the timing of flowering or flower and fruit production in the *cen4* background. An implication of this work is that kiwifruit *BFT* regulates bud dormancy. Thus, we provide tools for increasing understanding of the fundamentals of dormancy control and perhaps ultimately increasing productivity in warm climates with low winter chilling.

## Results

### *AcBFT2* is highly expressed in axillary buds during the period of no visible growth in winter and sharply increases in response to chilling

**Figure 1:**
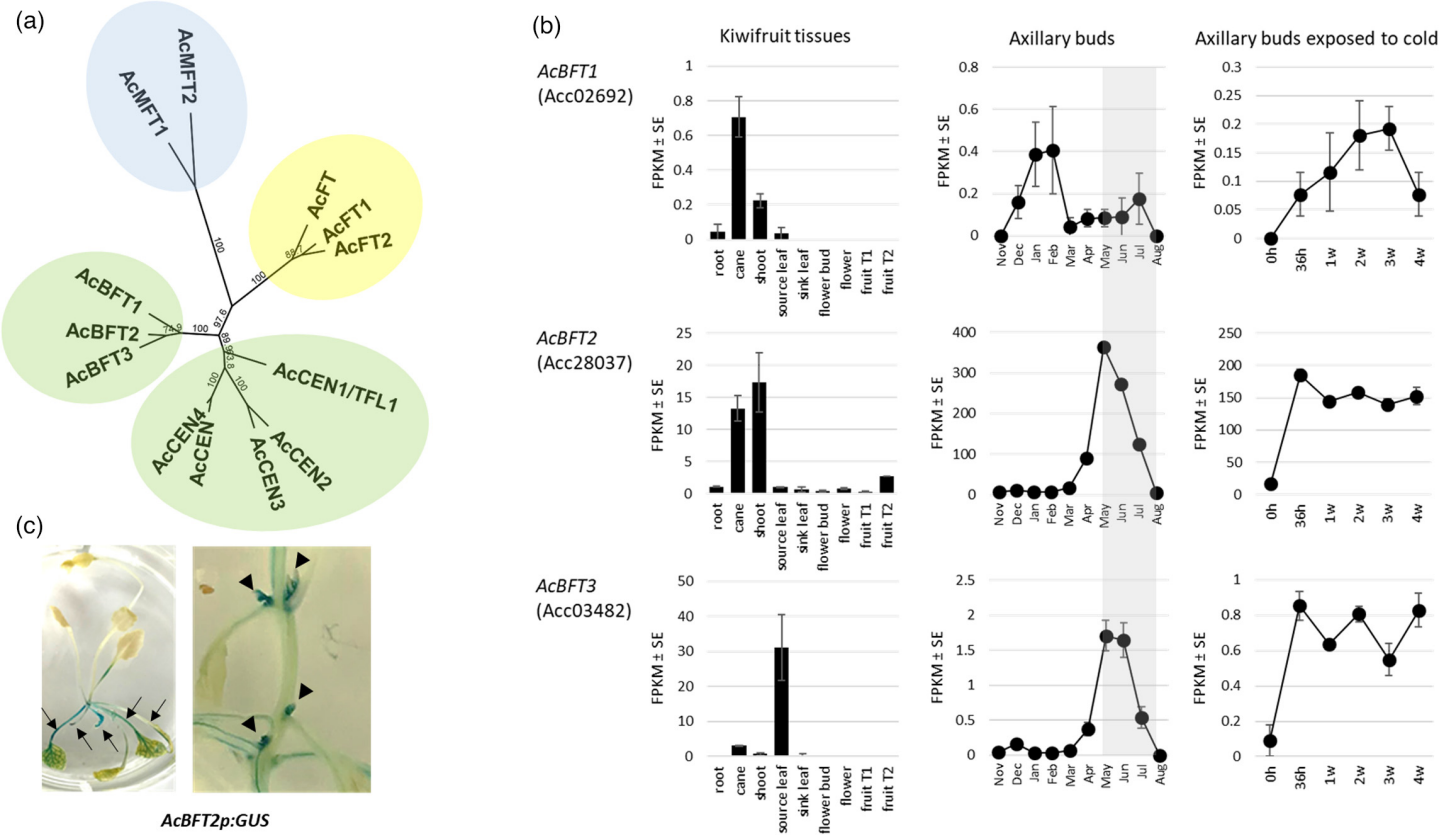
The kiwifruit PEBP gene family and gene expression. (a) A phylogenetic tree with the kiwifruit PEBP proteins. (b) RNA‐seq expression analysis of the three kiwifruit *BFT* genes in different kiwifruit organs, axillary buds collected from field‐grown plants at monthly intervals and axillary buds collected from excised canes exposed to cold over 4 weeks. The bars and circles represent mean FPKM ± SE of three biological replicates. The period of no visible growth during winter is shaded grey. Fruit T1 and T2 represent fruit at 20 and 40 days after anthesis respectively. (c) Localization of *AcBFT2* expression. A 2.5‐kb fragment upstream from the *AcBFT2* translational start site (*AcBFT2p*) was fused to *uidA* (GUS) and introduced into Arabidopsis. Histochemical localization of GUS activity in transgenic Arabidopsis identified promoter activity in the vascular tissue of cotyledons and rosette leaves (arrows, left) and axillary buds (arrowheads, right).

The kiwifruit PEBP gene family consists of 13 genes, encoding predicted proteins that separate into four lineages (Voogd *et al*., 2017) (Figures 1a, S1). Among the three AcBFT proteins, AcBFT2 and AcBFT3 are the most similar (89.6% identical), while they are 87.3% and 81.5% identical to AcBFT1. The three proteins (AcBFT1, AcBFT2 and AcBFT3) are in turn 71.3%, 68.4% and 67.2% identical to Arabidopsis BFT.

In our previously reported RNA‐seq datasets (Brian *et al*., 2021; Voogd *et al*., 2021), *AcBFT1* showed very low levels of expression in all tissues, including axillary buds collected each month from field‐grown vines over a yearly time course (Figure 1b). In contrast, *AcBFT2* transcripts accumulated in canes and shoots, and showed the highest levels in axillary buds (Figure 1b). *AcBFT2* transcript levels in axillary buds increased with growth cessation, peaked at the time of leaf drop and establishment of bud dormancy, and then declined with resumption of growth, as previously observed (Voogd *et al*., 2017). *AcBFT2* expression rapidly increased in axillary buds on excised canes exposed to an extended period of cold (Figure 1b).

While *AcBFT3* shared a similar pattern of expression to *AcBFT2* in the axillary bud time course and in excised canes exposed to cold, transcript levels were at considerably lower abundance (transcript accumulated to ~200 fold lower levels than those of *AcBFT2*). Amongst the different kiwifruit tissues tested, *AcBFT3* transcripts showed the greatest abundance in mature (source) leaves (Figure 1b).

To further investigate *AcBFT2* tissue‐specific expression, a transcriptional fusion of the 2.5‐kb fragment upstream from the *AcBFT2* translation start site with the reporter gene *uidA* (GUS) was introduced into Arabidopsis. GUS expression was detected in the vascular tissue of cotyledons and rosette leaves, but no staining was seen in cauline leaves, inflorescence stem or flowers. GUS staining was also detected in the stem vasculature and axillary buds of mature transgenic Arabidopsis (Figure 1c), demonstrating expression was confined to vascular tissue and buds, as previously shown for kiwifruit *FT* genes (Moss *et al*., 2018) and partly resembling the profile described for Arabidopsis *BFT* promoter fusion with GUS (Ryu *et al*., 2011). Like AcFT, AcBFT2 protein had the ability to interact with the kiwifruit FD protein, as detected by yeast two‐hybrid assays (Figure S2).

### Targeted CRISPR‐Cas9 mutagenesis of *AcBFT* genes with a focus on *AcBFT2*

**Figure 2:**
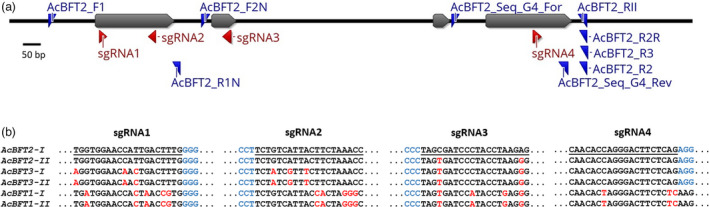
Design of the construct for CRISPR‐Cas9‐mediated mutagenesis of kiwifruit *AcBFT2*. (a) *AcBFT2* (GenBank accession number KX11601, gene model Acc28037) was used to design primers and single guide RNA (sgRNA) sequences. Exons are presented in grey, sgRNA target regions are indicated in red, and primer positions are indicated by blue arrowheads. (b) The sequences targeted by the gene editing construct. The target sequences used for sgRNAs are underlined, PAM sites are highlighted in blue, and mismatches between *AcBFT1, AcBFT2* and *AcBFT*3 alleles (I and II) and the sgRNAs are indicated in red.

The *AcBFT2* transcript showed high expression with a peak abundance in axillary buds that correlated with growth cessation and winter dormancy. To further study the role of *AcBFT* genes, particularly *AcBFT2*, CRISPR‐Cas9‐mediated gene editing was performed. We aimed to induce bi‐allelic editing of *AcBFT2*, while also potentially targeting *AcBFT3* (Figure 2a). Because of its nearly undetectable expression, we predicted that *AcBFT1* was unlikely to be functional, so it was not specifically targeted in this study (Figure 2b). Four guide sequences were designed based on the available published *A. chinensis BFT* sequences genome (Pilkington *et al*., 2018) (Figure 2b). SgRNA1 and sgRNA2 targeted exon 1 of *AcBFT2* specifically, while sgRNA3 and sgRNA4 targeted exons 2 and 4, respectively, in both *AcBFT2* and *AcBFT3* (Figure 2b). These were used to generate a polycistronic tRNA‐sgRNA construct, which was introduced into ‘Hort16A’ kiwifruit.

A total of 88 independent T0 transgenic lines carrying the *AcBFT* editing construct were produced and transferred to the glasshouse, in parallel with 10 control lines. Genotyping with *AcBFT2* allele‐specific primers confirmed bi‐allelic edits in *AcBFT2* in 23 lines and mutations affecting one of the *AcBFT2* alleles in 13 lines (Table 1). Only wild‐type *AcBFT2* sequences were identified in the remaining 52 lines, and these were maintained as non‐edited controls. Most of the mutations corresponded to sgRNA4 targeting exon 4, followed by sgRNA3 and sgRNA2. No mutations were identified in the sequence targeted by sgRNA1 in any of the lines. The mutations ranged from single nucleotide deletions to larger (>50 bp) deletions, or single nucleotide insertions occurring at different frequencies (Figure S3).

**Table 1:** Genotypes and phenotypes of *Acbft* and control kiwifruit lines in the ‘Hort16A’ background

Line ID	*AcBFT2*		AcBFT2 mutation type	*AcBFT3*		AcBFT3 mutation type	Phenotype		
	Allele I	Allele II		Allele I	Allele II				
	g1/g2/g3/g4	g1/g2/g3/g4		g1/g2/g3/g4	g1/g2/g3/g4		Year 1	Year 2	Year 3
*bft*#1	−/−/−/−2	−/−/−798	Bi‐allelic	n/t	n/t	n/t	evg	evg	evg
*bft*#9	−/−/−/−2	−/−/−791	Bi‐allelic	−/−/−/−	−/−/−/−4	Mono‐allelic	evg	evg	evg
*bft*#10	−/−/−/−2	−/−/−7/−3	Bi‐allelic	n/t	n/T	n/t	evg	evg	evg
*bft*#11	−/−/−/+1	−/−2/−6/+1	Bi‐allelic	−/−/−/−1	−/−/−/−2	Bi‐allelic	evg	evg	evg
*bft*#12	−/−/−2/−1	−/+1/−1/−12	Bi‐allelic	n/t	n/t	n/t	evg	evg	evg
*bft*#13	−/−/−2,+1/−1	−/+1/−1/−12	Bi‐allelic	n/t	n/t	n/t	evg	evg	evg
*bft*#14	−/−/−2,+1/−1	−/+1/−1/−12	Bi‐allelic	n/t	n/t	n/t	evg	evg	evg
*bft*#16	−/−/−2/−1	−/+1/−1/−12	Bi‐allelic	n/t	n/t	n/t	evg	evg	evg
*bft*#32	−/−/−5/−4	−/−/−1,+6/−8	Bi‐allelic	n/t	n/t	n/t	evg	evg	evg
*bft*#34	−/−400/−4	−/−/−/+7	Bi‐allelic	n/t	n/t	n/t	evg	evg	evg
*bft*#39	−/−/−/−30	−/−/−/−4	Bi‐allelic	n/t	n/t	n/t	evg	evg	evg
*bft*#43	−/−/−/−30	−/−/−/+1	Bi‐allelic	n/t	n/t	n/t	evg	evg	evg
*bft*#52	−/−/−/−2	−/−/−791	Bi‐allelic	n/t	n/t	n/t	evg	evg	evg
*bft*#55	−/−4/−2/−3	−/−4/−/−	Bi‐allelic	n/t	n/t	n/t	evg	evg	evg
*bft*#56	−/−/−/+1	−/−/−/+1	Bi‐allelic	n/t	n/t	n/t	evg	evg	evg
*bft*#58	−/−/−14/−4	−/−/−/+2	Bi‐allelic	n/t	n/t	n/t	evg	evg	evg
*bft*#59	−/−/−/−2	−/−/−1/+1	Bi‐allelic	n/t	n/t	n/t	evg	evg	evg
*bft*#61	−/−/−/−2	−/−/−799	Bi‐allelic	n/t	n/t	n/t	evg	evg	evg
*bft*#65	−/−/−/+1	−/−/−/−11	Bi‐allelic	n/t	n/t	n/t	evg	evg	evg
*bft*#70	−/−/−/−4	−/−/−/+1	Bi‐allelic	n/t	n/t	n/t	evg	evg	evg
*bft*#72	−/−/−/+1	−/−/−1/−2	Bi‐allelic	n/t	n/t	n/t	evg	evg	evg
*bft*#79	−/−/−/+1	−/−/−13/−17	Bi‐allelic	n/t	n/t	n/t	evg	evg	evg
*bft*#81	−/−/−9/−2	−/−/−1/−2	Bi‐allelic	n/t	n/t	n/t	evg	evg	evg
*bft*#3	−/−/−/+1	−/−/−/−	Mono‐allelic	−/−/−/−5	−/−/−/−3	Bi‐allelic	C	C	C
*bft*#41	−/−/−/−30	−/−/−/−	Mono‐allelic	−/−/−/−3	−/−/−/−1	Bi‐allelic	C	C	C
*bft*#6	−/−/−/−	−/−/−/−	WT	−/−/−/−	−/−/−/−2	Mono‐allelic	C	C	C
*bft*#4	−/−/−/−	−/−/−/−	WT	−/−/−/−	−/−/−/−	WT	C	C	C
*bft*#8	−/−/−/−	−/−/−/−	WT	−/−/−/−	−/−/−/−	WT	C	C	C
*bft*#15	−/−/−/−	−/−/−/−	WT	−/−/−/−	−/−/−/−	WT	C	C	C
*bft*#18	−/−/−/−	−/−/−/−	WT	−/−/−/−	−/−/−/−	WT	C	C	C
*bft*#21	−/−/−/−	−/−/−/−	WT	−/−/−/−	−/−/−/−	WT	C	C	C
*bft*#23	−/−/−/−	−/−/−/−	WT	−/−/−/−	−/−/−/−	WT	C	C	C
*bft*#24	−/−/−/−	−/−/−/−	WT	−/−/−/−	−/−/−/−	WT	C	C	C
*bft*#25	−/−/−/−	−/−/−/−	WT	−/−/−/−	−/−/−/−	WT	C	C	C

Comparison to wild‐type (WT) sequence identified deletions (−n), insertions (+n) or no change (−) in positions targeted by single guide RNA sequences g1‐g4. n/t, not tested; evg, evergrowing phenotype (delayed leaf drop and budset, earlier budbreak than controls); C, control phenotype (growth cessation, leaf drop and budbreak comparable to control lines).

Thirteen lines were subjected to further genotyping for edits in *AcBFT3*. Additional edits of one or both alleles of *AcBFT3* were identified in lines with bi‐allelic *AcBFT2* mutations. Two analysed lines had edits in a single *AcBFT2* and both *AcBFT3* alleles. However, lines with wild‐type *AcBFT2* sequences also had no edits in *AcBFT3* alleles, except one line with edits in only one of the *AcBFT3* alleles (Table 1). Therefore, as none of the transgenic lines we analysed carried bi‐allelic mutations specific to *AcBFT2* or *AcBFT3* only, we refer to them as *Acbft* mutants.

### Delayed dormancy and early budbreak in *Acbft* mutant lines results in an evergrowing phenotype

**Figure 3:**
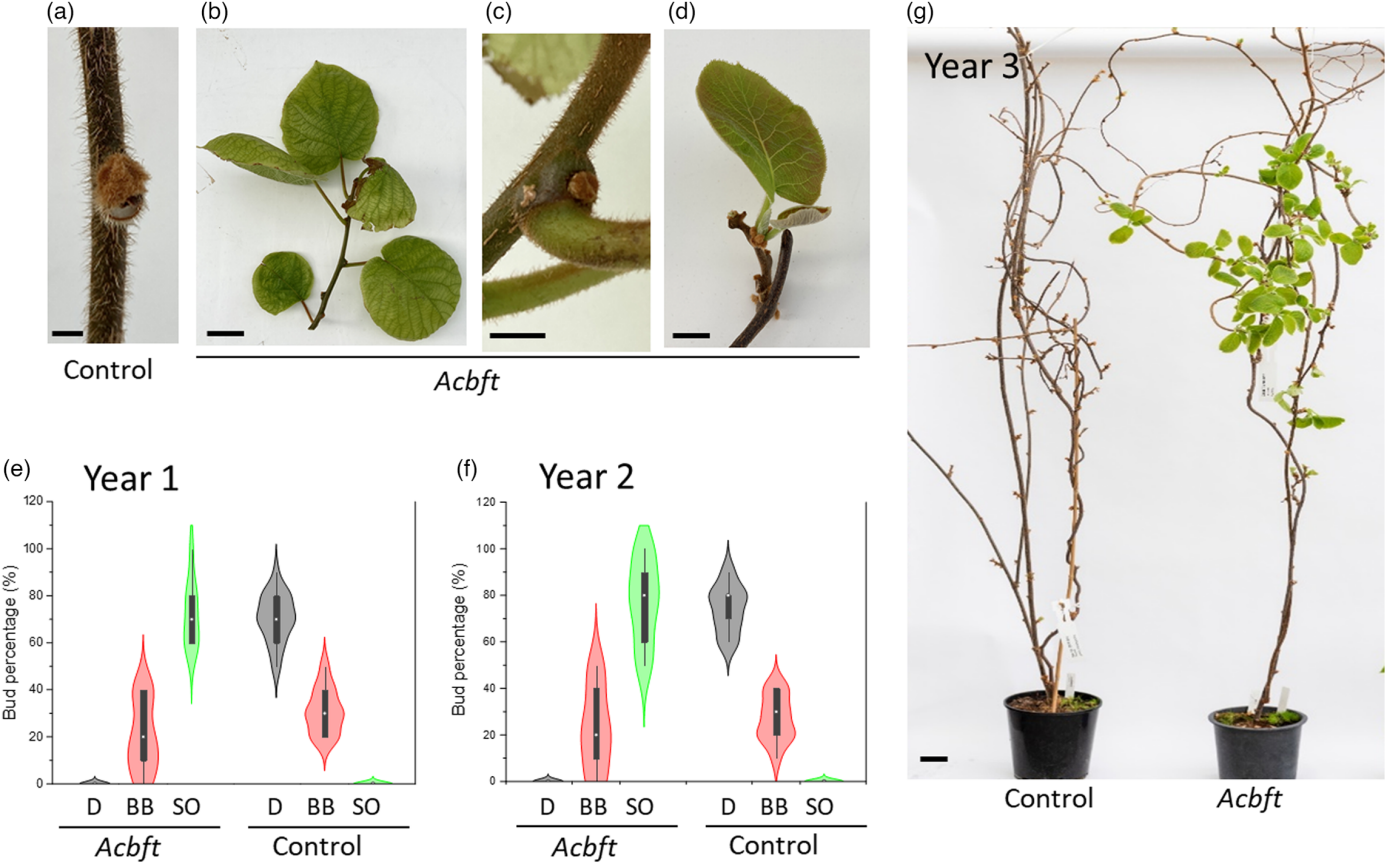
Delayed dormancy and early budbreak in *Acbft* kiwifruit lines. (a) A dormant bud in a control line. (b–d) Examples of delayed leaf senescence (b), delayed budset (c), and emergence of new shoots (d) in *Acbft* lines. Photographs (a–d) taken on 11 July 2021. (e, f) Early budbreak in *Acbft* lines. Frequency of dormant buds (D), budbreak (BB) and shoot outgrowth (SO) in *Acbft* and control plants. The plants were defoliated and data were recorded after 4 weeks using 20 distal buds from 18 lines with bi‐allelic edits in *AcBFT2* (*Acbft*) and 20 control and non‐edited lines (Control). The white dot is the median, the black bar is the interquartile range, the whiskers represent the minimum and maximum, and the violin represents the distribution of the data. (g) Earlier budbreak of a representative *Acbft* edited line (right) compared with the control (left) in year 3. Photograph taken on 17 August 2021. Scale bars represent 5 cm.

Gene‐edited *Acbft* and control lines grown in the glasshouse were monitored over 3 years (2019, 2020 and 2021). Each year, autumn leaf senescence was followed by substantial natural abscission and budset in control lines, with most leaves dropping and all buds displaying a dormant appearance in winter (Figure 3a). Strikingly, much less growth cessation and senescence were seen in the *Acbft* lines with bi‐allelic edits in *AcBFT2*. While some buds appeared dormant, shoots with mostly green leaves, lack of budset and sporadic shoot emergence from distal buds were observed, producing an evergrowing phenotype (Figure 3b–d). Phenotypes of lines with edits in only one *AcBFT2* allele were comparable to controls (Table 1). Therefore, the loss of *AcBFT2* function caused by bi‐allelic edits probably underpins the delay in growth cessation, onset of leaf senescence and budset in these *Acbft* lines.

To further evaluate the role of *AcBFT* genes in regulation of budbreak in kiwifruit, *Acbft*, control and non‐edited plants were pruned each year in June to a similar size and defoliated. After defoliation, buds on all plants appeared dormant, but visible growth quickly resumed in the *Acbft* lines with bi‐allelic *AcBFT2* edits (Figures 3e–g, S4, Table 1). Within a period of 4 weeks from defoliation (in July), scoring of distal buds across edited and control lines identified shoot outgrowth and very few dormant buds in edited lines (Figure 3e,f). In contrast, the appearance of controls and non‐edited lines was mostly dormant, with little visible budbreak (Figure 3e,f) and no or little growth observed after an additional period of 4 weeks, in August (Figures 3g, S4). Budbreak comparable to that in controls was also found in lines with mutations in a single *AcBFT2* allele, in spite of additional edits in *AcBFT3* (Figure S4, Table 1).

### *AcBFT* affects kiwifruit architecture but not reproductive maturity and flowering

**Figure 4:**
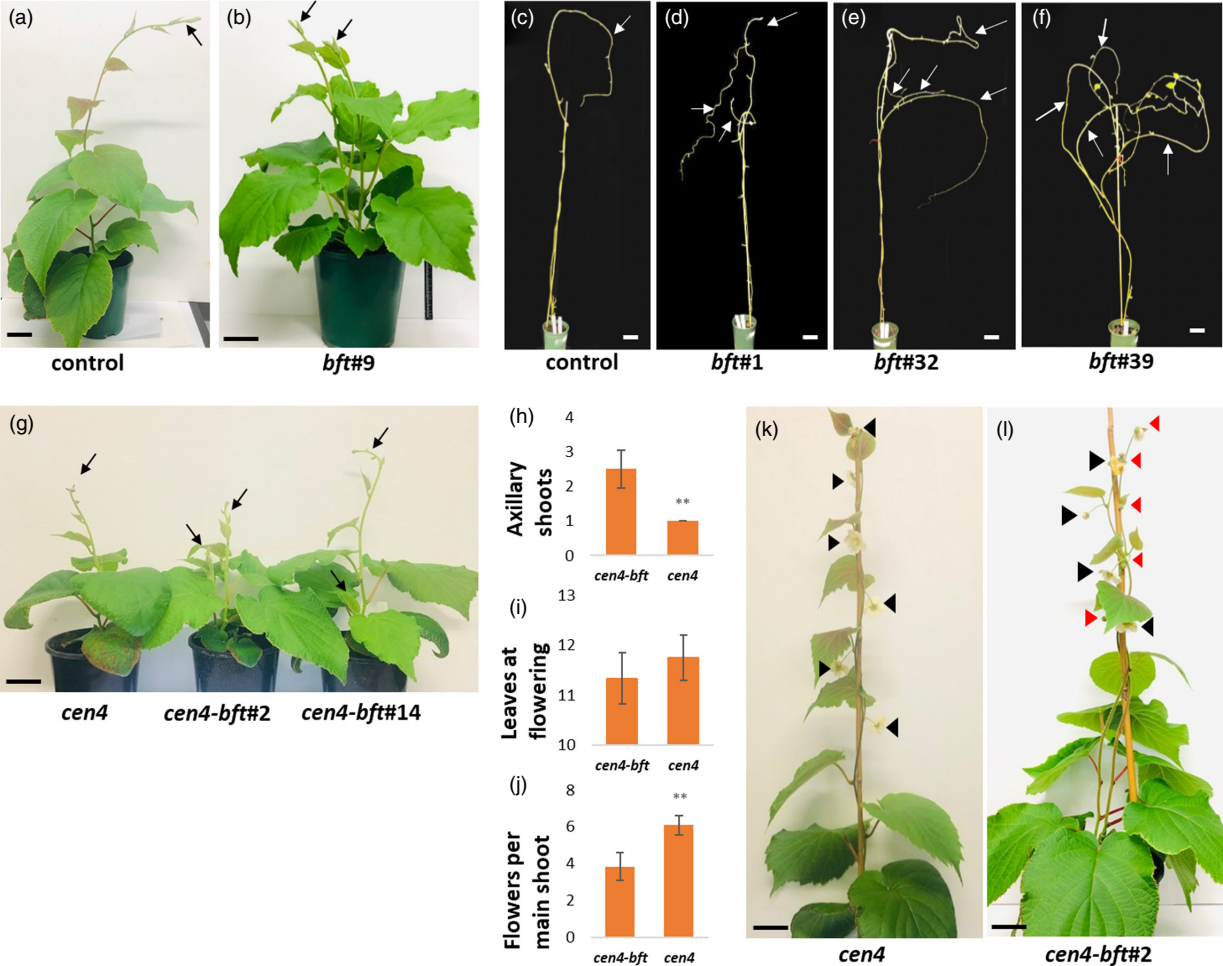
*AcBFT* regulates shoot architecture in kiwifruit. (a, b) Vigorous main shoot in a representative control and multiple shoots in a representative *Acbft* kiwifruit mutant line (*bft*#9) after establishment in the glasshouse. Shoots are indicated with black arrows. (c–f) Architecture of kiwifruit lines after first year's growth. The plants have been defoliated. Branches are indicated with white arrows. (g) Branching on representative fast‐flowering U6‐CEN4#7 (*cen4*) and double *Acbft cen4* mutant lines (*cen4‐bft*). Shoots are indicated with black arrows. (h) The number of axillary shoots on *cen4* and double mutant lines. (i, j) The numbers of leaves and flowers on the main (longest) shoot of *cen4* and double mutant plants. The bars represent a mean ± SE of 6 *cen4‐bft* and 13 *cen4* lines. **Indicates statistical significance (*P* < 0.05). (k, l) Appearance of flowers. Flowers on the main (longest) shoot are indicated with black arrowheads. Orange arrowheads mark flowers on the side shoot. Scale bars represent 5 cm.

The general appearance of *Acbft* gene‐edited lines also suggested an increase in branching, with more axillary shoots observed than in controls. Upon transfer to the soil, the control lines displayed the usual kiwifruit growth pattern, with a rapidly growing single main shoot. In edited lines, concomitant development of two or three shoots was seen (Figure 4a,b) and unpruned plants commonly had multiple branches by the first year's winter (Figure 4c–f). However, *Acbft* plants were no more compact than controls and none of the *Acbft* lines flowered over three seasons (2019–2022), indicating that *Acbft* mutations do not accelerate reproductive maturity, unlike mutations in *AcCEN*/*AcCEN4* (Varkonyi‐Gasic *et al*., 2019).

To further investigate any effects on flowering, editing of *AcBFT2* was also carried out in the fast‐flowering kiwifruit *cen4* mutant (U6‐CEN4#7). Six *cen4* plants were identified that had bi‐allelic edits in the *AcBFT2* gene and four with mono‐allelic mutations (Table 2). Additional branching was again observed in the double mutant *Acbft cen4* lines relative to the *cen4* mutant (Figure 4g,h). However, mutation of *Acbft2* in the early‐flowering *cen4* background did not have an effect on flowering time. A terminal flower was produced after 11–12 leaves in the *cen4* lines and in the double *Acbft cen4* mutant, indicating no additive effects of the *Acbft2* mutation (Figure 4i). Initially, a small reduction in the number of axillary flowers on the main shoot was seen in the double mutant lines (Figure 4j); however, all shoots were floral (Figure 4k,l, Table 2). The morphology of the flowers was not affected and pollination resulted in the development of fertile fruit (Figure S5).

**Table 2:** Genotypes and phenotypes of *Acbft cen4* lines. Comparison to wild‐type (WT) sequence identified deletions (−n), insertions (+n) or no change (−) in positions targeted by single guide RNA sequences g1‐g4.

Line ID	*AcBFT2*		AcBFT2 mutation type	# of shoots	# of floral shoots
	allele I	allele II			
	g1/g2/g3/g4	g1/g2/g3/g4			
*cen4‐bft*#2	−/−/−/−2	−/−/−/+1	Bi‐allelic	2	2
*cen4‐bft*#14	−/−/−/−2	−/−/−1/−2	Bi‐allelic	3	3
*cen4‐bft*#15	−/−/−/−1	−/−/−1/−2	Bi‐allelic	3	3
*cen4‐bft*#18	−/−/−/−1	−/−/−1/−2	Bi‐allelic	3	3
*cen4‐bft*#20	−/−/−1/−2	−/−/−/−1	Bi‐allelic	2	2
*cen4‐bft*#21	−/−/−/−1	−/−/−/−2	Bi‐allelic	2	2
*cen4‐bft*#6	−/−1/−/−	−/−/−/−	Mono‐allelic	2	2
*cen4‐bft*#10	−/−1/−/−	−/−/−/−	Mono‐allelic	1	1
*cen4‐bft*#19	−/−/−/−2	−/−/−/−	Mono‐allelic	1	1
*cen4‐bft*#23	−/−/−1/−2	−/−/−/−	Mono‐allelic	1	1
*cen4‐bft*#3	−/−/−/−	−/−/−/−	WT	1	1
*cen4‐bft*#4	−/−/−/−	−/−/−/−	WT	1	1
*cen4‐bft*#5	−/−/−/−	−/−/−/−	WT	1	1
*cen4‐bft*#7	−/−/−/−	−/−/−/−	WT	1	1
*cen4‐bft*#8	−/−/−/−	−/−/−/−	WT	1	1
*cen4‐bft*#9	−/−/−/−	−/−/−/−	WT	1	1
*cen4‐bft*#11	−/−/−/−	−/−/−/−	WT	1	1
*cen4‐bft*#12	−/−/−/−	−/−/−/−	WT	1	1
*cen4‐bft*#13	−/−/−/−	−/−/−/−	WT	1	1
*cen4‐bft*#16	−/−/−/−	−/−/−/−	WT	1	1
*cen4‐bft*#17	−/−/−/−	−/−/−/−	WT	1	1
*cen4‐bft*#22	−/−/−/−	−/−/−/−	WT	1	1
*cen4‐bft*#24	−/−/−/−	−/−/−/−	WT	1	1

The control *cen4* U6‐CEN4#7 lines produced a single floral shoot as described in Varkonyi‐Gasic *et al*. (2019).

### RNA‐seq analysis of *Acbft* buds

**Figure 5:**
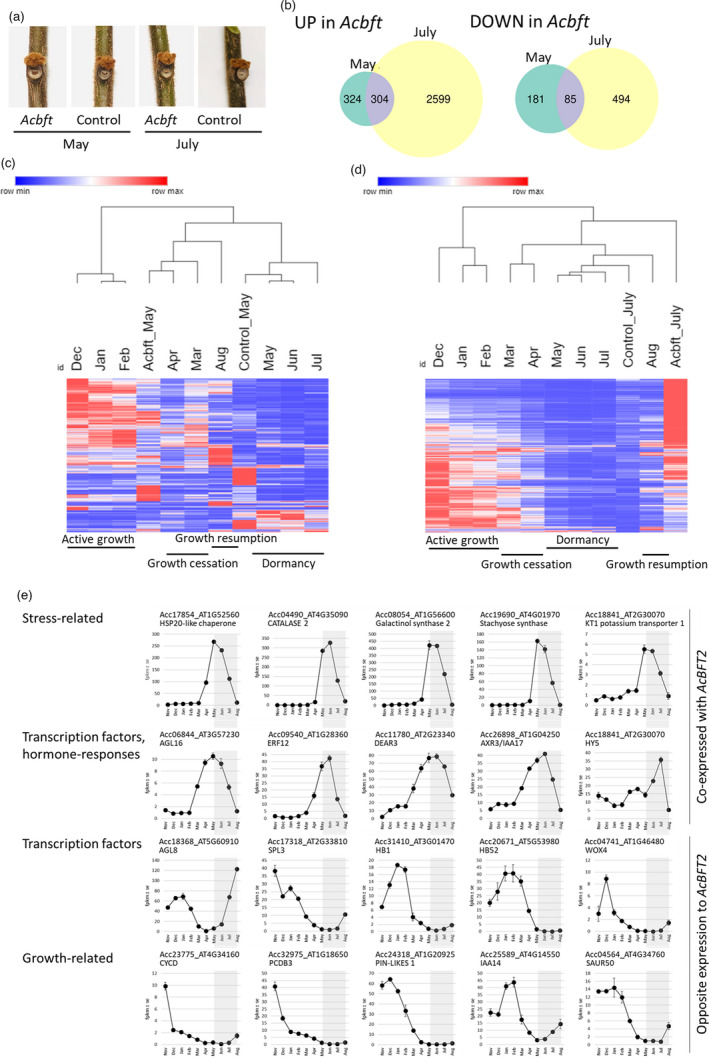
Transcriptome analysis. (a) Representative kiwifruit bud samples. (b) Venn diagrams of differentially expressed genes (DEGs) in *Acbft* mutants relative to controls in May and July. (c, d) Hierarchical clustering (One minus Pearson's correlation, average linkage, using mean FPKMs of three biological replicates) of gene expression of DEGs in May (c) and July (d) with gene expression in wild‐type buds collected in different months in the field. The phenological stages of wild‐type buds are indicated below. (e, f) Examples of genes identified as co‐expressed with *AcBFT2* (e) or with expression opposite to that of *AcBFT2* (f). The graphs represent expression (mean FPKM ± SE of three biological replicates) of different classes of candidate genes in axillary buds collected from field‐grown plants at monthly intervals. The kiwifruit gene identification and closest Arabidopsis homologue and its function are indicated.

To investigate the molecular mechanisms of *AcBFT* action, RNA‐seq transcriptome analysis of buds collected from *Acbft* and control lines at two time points (May and July, 2020) was performed (Table S1). Three *Acbft* lines with bi‐allelic *AcBFT2* edits were chosen and compared with controls, using buds with similar visible morphology (Figure 5a).

Analysis of bud transcriptomes collected in autumn (May), identified 894 differentially expressed genes (DEGs) with the minimum of twofold change in expression (DESeq, adjusted *P*‐value < 0.05, FPKM >1) between *Acbft* and control lines (Table S2). Of those, 628 and 266 were up‐ and down‐regulated, respectively, in *Acbft* lines in May (Figure 5b). A subset of these DEGs remained up‐ and down‐regulated in July (304 and 85 respectively), with a further 2599 genes showing increased and 494 genes showing decreased expression in July, with at least twofold change in expression (Figure 5b, Table S3).

Hierarchical clustering demonstrated a clear separation between the *Acbft* and control lines (Figure 5c,d). Comparison with transcriptomes of buds across phenological stages collected from field‐grown plants (Brian *et al*., 2021; Voogd *et al*., 2021) grouped the *Acbft* bud DEGs in May with wild‐type samples from earlier in the year (April), while as expected a correlation with winter dormant buds (May–June) was seen for the controls (Figure 5c). The apparent difference in the dormancy status between the buds was further supported by the predicted function of DEGs. GO‐term categories related to abscisic acid (ABA) and gibberellic acid (GA) metabolism and signalling, auxin polar transport, growth and morphogenesis and vascular transport were enriched amongst up genes in *Acbft* buds in May (Figure S6a). Genes down in *Acbft* displayed enrichment in stress‐related categories (Figure S6a). For the July‐sample DEGs, the control was placed within the group with little active growth, while a large proportion of *Acbft* DEGs were highly up‐regulated compared with all other samples (Figure 5d). Enrichment in DNA replication, cell cycle and cell division‐related categories corresponding to resumption of active growth was seen in DEGs up‐regulated in *Acbft* buds in July (Figure S6b). In contrast, temperature, osmotic and other stress response categories were enriched amongst the 579 genes down‐regulated in *Acbft* relative to controls (Figure S6b).

The subsets of genes consistently up‐ or down‐regulated in *Acbft* mutants, regardless of sampling date (Figure 5b, Table S4) were subjected to further analysis. They represent genes likely to be regulated by the presence of *AcBFT* in buds. There were 304 and 85 genes that were always up‐ or down‐regulated, respectively, in *Acbft* lines compared with controls (Figure 5b). As expected, *AcBFT2* itself was one of these down‐regulated genes in *Acbft* mutants, but not *AcBFT1* and *AcBFT3*. The set of 85 genes with low expression in the absence of *AcBFT* contained genes encoding for temperature, oxygen and other stress‐response proteins such as catalases, peroxidases, heat shock proteins and enzymes producing osmoprotectants and transporters (Table S4). Genes encoding transcription factors and hormone response genes were also identified, including a MADS‐box gene with homology to AGL16, AP2/ERF family transcription factors, and two bZIP *HY5* homologues (Table S4). Examination of these down‐regulated transcription factors in *Acbft* in yearly expression profiles of field‐grown wild‐type buds across phenological stages (Brian *et al*., 2021; Voogd *et al*., 2021) indicated that some of them showed a pattern similar to the expression to *AcBFT2*, consistent with them being able to mediate *AcBFT* function, by interacting with AcBFT, or acting upstream or downstream of *AcBFT* itself (Figure 5e).

Furthermore, DEGs whose expression increased in the absence of *AcBFT2* were considered as possible targets of AcBFT‐mediated repression. Amongst the 304 genes that were always up‐regulated in *Acbft* mutants regardless of the sampling date (Figure 5b), there were multiple *CYCLIN* and *EXPANSIN* homologues, possibly marking cell divisions and expansion, and genes encoding transcription factors implicated in different aspects of differentiation and development, for example WOX and TCP, and with growth or flowering, such as *SPL*, *CYCLING DOF FACTORS* and *FRUITFUL*/*AGL8* homologues (Table S4), possibly contributing to the evergrowing phenotype. Many of these genes showed a pattern opposite to *AcBFT2* in wild‐type buds sampled across phenological stages, with no or low expression during the dormancy period (Figure 5e). None of the three kiwifruit *FT* genes was identified as differentially expressed, and very low expression (<1 FPKMs) was detected in all the samples (Table S1). Similarly, no or very low expression (FPKM <5) was detected for kiwifruit PEBP genes other than *AcBFT2* (FPKM >40). *AcCEN* was identified in *Acbft* buds in July, while *AcBFT1* and *AcCEN2* could be detected in May in *Acbft* and control buds respectively (Tables S2‐S4). Elevated *AcFD* (Acc05237) in May compared with July was detected in both edited and control lines, while *AcFD* expression declined in kiwifruit buds during dormancy and cold treatment (Figure S7).

## Discussion

### *Acbft* kiwifruit mutants do not have altered flowering

Members of the PEBP gene family are present in all eukaryotes and are central to plant development and physiology. Yet, in contrast to the pivotal roles *FT* and *TFL1* genes play in Arabidopsis architecture and flowering, *BFT* appears to have a relatively minor role in contributing to flowering time control and to regulation of Arabidopsis axillary inflorescence development under stress conditions (Chung *et al*., 2010; Ryu *et al*., 2011; Yoo *et al*., 2010), through competition with FT for binding to FD (Ryu *et al*., 2014).

The *Actinidia* PEBP family contains multiple members, arising from recent whole‐genome duplication events (Huang *et al*., 2013), with the *BFT* lineage consisting of three genes. *AcBFT2* and *AcBFT3* have high similarity in sequence, but differential expression in stem and leaf tissues and distinctively high accumulation of *AcBFT2* transcript in dormant buds. In contrast, *AcBFT1* is more divergent and is barely expressed. The three AcBFT proteins are predicted to have one or both the conserved His and Asp residues responsible for floral repressor activity of TFL1 (Ahn *et al*., 2006; Hanzawa *et al*., 2005). Indeed, a delay in bolting combined with changed inflorescence architecture was demonstrated upon expression of *AcBFT* genes in Arabidopsis, implicating the *AcBFT* family in repression of flowering in a similar manner to *AcCEN* and *AcCEN4* (Voogd *et al*., 2017). In particular, a compact, rapid‐cycling kiwifruit produced by gene editing of the *CEN* genes (*AcCEN* and *AcCEN4*) demonstrated the role of these genes as central repressors of the onset of the reproductive phase.

However, we show here that *AcBFT* genes are not likely to regulate flowering in kiwifruit. In contrast to the short juvenility, compact size and continuous flowering of *cen* or *cen4* mutants (Varkonyi‐Gasic *et al*., 2019), the loss of *AcBFT* did not affect reproductive maturity and plant size in gene‐edited kiwifruit plants. Furthermore, editing of *AcBFT* in the rapid flowering *cen4* background did not impact flowering time, similarly to the *bft tfl1* double mutant, but unlike the *tfl atc bft* triple mutant in Arabidopsis (Kim *et al*., 2013; Yoo *et al*., 2010), or the faster flowering double *cen4 cen* kiwifruit mutant (Varkonyi‐Gasic *et al*., 2019). A minor reduction in flower numbers was observed on the main shoot, which could be interpreted as a competition for resources in flower *vs* axillary shoot development. This finding was comparable to the accelerated termination of primary inflorescence (manifested through reduction in solitary flower numbers) and precocious development of axillary inflorescences in the absence of BFT function in Arabidopsis (Yoo *et al*., 2010). However, all shoots of double mutants were floral and there were no adverse effects on fruit development or fertility.

### BFT controls growth cessation and dormancy

In temperate perennial trees and vines such as kiwifruit, the timing of dormancy onset and release is highly regulated to enable survival of unfavourable conditions and maximize production in the following growing season. Autumn conditions initiate growth cessation, preparing plants for the harsh winter conditions. In turn, winter chilling accumulation is required to break dormancy and initiate flowering (Lionakis and Schwabe, 1984a,b). Warming winter temperatures pose a large risk to kiwifruit productivity. Understanding the molecular regulation of kiwifruit phenology, with a particular focus on seasonally controlled cycles of growth and dormancy, is essential for development and selection of low‐chill varieties.

Here, editing of *AcBFT* demonstrated an important role for these genes in promoting the onset of dormancy. This is consistent with recent findings suggesting that PEBP proteins integrate various environmental conditions with internal sink and source transitions, to initiate flowering (Andrés *et al*., 2020) or outgrowth of different organs, for example, underground storage organs (Abelenda *et al*., 2019) that enable survival and ultimately, reproduction. AcBFT performs a similar pivotal role as a regulator of kiwifruit bud phenology, thus controlling its temperate perennial life history and reproduction strategy.

Because of the high sequence similarity between *AcBFT* genes and alleles, we were unable to design gene‐specific editing guides. Lines with edits in both alleles of *AcBFT2* demonstrated an evergrowing phenotype, suggesting a role for *AcBFT2* in seasonal growth cessation and establishment of winter dormancy, consistent with *AcBFT2* accumulation in dormant buds (Voogd *et al*., 2017). However, lines with bi‐allelic mutations in *AcBFT2* locus contained additional edits in *AcBFT3*. Therefore, at this stage, it remains unclear if bi‐allelic editing of *AcBFT2* only is sufficient for the evergrowing phenotype. We were also unable to fully exclude possible edits in *AcBFT1*. Despite the very low expression in various tissues, a possibility exists that *AcBFT1* is regulated in a highly specific temporal manner and accumulates in very specific cells, contributing to the regulation of growth and dormancy cycles. Therefore, edits in both alleles in *AcBFT2*, with the possible contribution of mutations in other *AcBFT* loci, cause continual growth in autumn and promote budbreak upon defoliation in winter. Nevertheless, evidence from two lines demonstrated that bi‐allelic mutations in *AcBFT3*, even in a combination with a mutation in one of the *AcBFT2* alleles, are insufficient for continual shoot growth in winter. Taken together and combined with elevated *AcBFT2* but not *AcBFT1* and *AcBFT3* in dormant buds, we conclude that *AcBFT2* acts to repress growth in dormancy‐inducing conditions.

This is comparable to the *BFT* role in axillary inflorescence development under stress conditions in Arabidopsis (Chung *et al*., 2010; Ryu *et al*., 2011, 2014) and consistent with findings that PEBP genes regulate seasonal growth in woody perennials (Böhlenius *et al*., 2006; Hsu *et al*., 2011; Miskolczi *et al*., 2019; Mohamed *et al*., 2010; Rinne *et al*., 2011). Some *BFT*‐like genes from woody perennial plants have also been clearly associated with expression in buds and stems, resembling *AcBFT2*. For example, the grape *BFT*‐like *VvTFL1c* transcript accumulates during latent bud development (Carmona *et al*., 2007). Expression in stems was also reported in apple, with two *BFT* transcripts highly enriched in dwarfing rootstocks (Foster *et al*., 2013), implicating *BFT* in reduced scion vigour accompanied with precocity.

Recently, expression of *BFT* was detected in Arabidopsis leaf, correlating with bolting and onset of senescence (Hinckley and Brusslan, 2020), which may be similar to our observation of elevated *AcBFT3* in mature, source leaves, as well as GUS profiles obtained with *AcBFT2* promoter in Arabidopsis. Accumulation in stem and leaf combined with expression in vascular tissue brings about the possibility that *BFT* genes may exert their function outside the domains of their expression, either through activation of a mobile signal, inactivation of another mobile signal, or translocation of their own protein. Non‐cell autonomous activity over long distances has been reported for FT florigen (Corbesier *et al*., 2007; Lifschitz *et al*., 2006; Lin *et al*., 2007) and more recently in regulation of vegetative phenology in poplar (Miskolczi *et al*., 2019), while TFL1 moves within cell layers of the shoot apical meristem (Conti and Bradley, 2007; Goretti *et al*., 2020), but future work will be needed to address if either of these scenarios applies to AcBFT. The mechanism of AcBFT action may include an interaction with an FD transcription factor AcFD; however, it remains unclear if kiwifruit BFT proteins exert their function through competing with other PEBP proteins. For example none of the *FT* genes was expressed in the buds, and *FT* expression in juvenile plants is very low (Varkonyi‐Gasic *et al*., 2013), but this may not reflect FT protein activity. Similarly, *AcFD* is expressed during active growth and down‐regulated in dormant buds when *AcBFT2* accumulation peaks. It is possible that the accumulation and removal of BFT activity may be sufficient to regulate bud phenology in kiwifruit independently of FT. This may be similar to kiwifruit *CEN* genes, whose removal by CRISPR‐Cas9 editing was sufficient to induce precocious flowering (Varkonyi‐Gasic *et al*., 2019).

Loss of *AcBFT* and the resulting evergrowing phenotype were accompanied by down‐regulation of homologues of transcription factors such as AP2/ERFs, acting as key participants in stress and hormone responses (reviewed in Xie *et al*., 2019), HY5 with many roles in plant growth and development (reviewed in Gangappa and Botto, 2016) and AGL16, which is a negative regulator of salt stress response and a flowering repressor, interacting with SVP and FLC to down‐regulate *FT* (Szaker *et al*., 2019; Zhao *et al*., 2021). In contrast, expression opposite to that of *AcBFT2* was shown for multiple regulatory genes and transcription factors implicated in growth. The predicted function of these genes, combined with an expression similar or opposite to that of *AcBFT2*, is consistent with roles in the dormancy regulatory networks in which *AcBFT* plays a central role, as demonstrated by mutagenesis in this study.

In summary, editing of kiwifruit *BFT* results in an evergrowing habit and considerably reduces the chilling requirement for spring budbreak, providing the opportunity to grow kiwifruit in regions with insufficient chilling without the application of dormancy‐breaking chemicals. The generated lines provide a tool to understand the molecular regulation of seasonal growth and dormancy cycles in kiwifruit. Furthermore, gene editing of *BFT* genes could accelerate the development of new low‐chill varieties across the *Actinidia* genus.

## Materials and methods

### Plant material

Stock plants of *Actinidia chinensis* (kiwifruit) Planch. var. *chinensis* ‘Hort16A’ and a fast‐flowering *cen4*‐edited ‘Hort16A’ line, U6‐CEN4#7 (Varkonyi‐Gasic *et al*., 2019) used in transformation experiments, were obtained from *in vitro* collections held at Plant & Food Research, Auckland, New Zealand. For kiwifruit gene expression graphs, the samples and RNA‐seq analysis have been described previously (Brian *et al*., 2021; Voogd *et al*., 2021). The RNA‐seq of *Acbft* mutants and control plants was performed with bud material collected from 2‐year‐old transgenic plants in winter (May and July 2020). Arabidopsis Col‐0 was used for transformation of the promoter fusion construct.

### Vector construction

The construct for CRISPR‐Cas9‐mediated mutagenesis was designed to contain a polycistronic tRNA‐sgRNA cassette with four sgRNA sequences, placed under the control of the Arabidopsis U6‐26 promoter. Suitable targets within the *AcBFT2* locus were identified using the Geneious 10.0.9 (https://www.geneious.com) CRISPR selection tool, applying the described quality scoring (Doench *et al*., 2014). The entry clone pENTR‐pAtU6.26‐BFT‐g1.g2.g3.g4 was constructed using assembly of Golden Gate parts amplified by PCR as described by Voogd *et al*. (2021). This entry clone was subsequently cloned into pDE‐KRS (Varkonyi‐Gasic *et al*., 2019) and pDE‐KRS‐HYG (Akagi *et al*., 2019) by Gateway cloning to produce pDE‐KRS‐pAtU6.26‐BFT‐g1.g2.g3.g4 (kanamycin resistance) and pDE‐KRS‐HYG‐pAtU6.26‐BFT‐g1.g2.g3.g4 (hygromycin resistance). To generate control transgenic lines, the pDE‐KRS‐pAtU6.26‐GFP‐g1 vector was used, containing a single sgRNA targeting the green fluorescent protein (GFP) sequence. This plasmid was constructed by exchanging the NheI fragment in pDE‐KRS‐pAtU6.26‐T2_AcCEN4 (described below) by an NheI‐digested PCR fragment produced by overlap PCR using pDE‐KRS‐specific primers and tRNA‐sgRNA cassette‐specific primers flanked by GFP sequence overhangs creating the GFP target. The pDE‐KRS‐pAtU6.26‐T2_AcCEN4 plasmid was constructed similarly to the pDE‐KRS‐pAtU6.26‐BFT‐g1.g2.g3.g4 construct. All oligonucleotide primers are listed in Table S5.

For the promoter fusion construct AcBFT2p:GUS, a 2.5‐kb fragment upstream from the *AcBFT2* translation start site was amplified from kiwifruit ‘Hort16A’ genomic DNA, re‐amplified to introduce restriction sites and cloned as an AscI/KpnI fragment to generate a transcriptional fusion with the reporter gene uidA (GUS) in the pHEX vector (Moss *et al*., 2018). All constructs were introduced by electroporation into *Agrobacterium tumefaciens* strain EHA105 for kiwifruit transformation, and GV3101 for Arabidopsis transformation.

For the yeast two‐hybrid assay, *AcBFT2* cloned into pDONR221 vector (Voogd *et al*., 2017) was recombined into pDEST32 (pBDGAL4, bait) and pDEST22 (pADGAL4, prey; Invitrogen) according to the manufacturer's instructions. *AcFT* and *AcFD* constructs were as previously described (Varkonyi‐Gasic *et al*., 2013). Bait and prey constructs were transformed into yeast strains PJ69‐4α and PJ69‐4a respectively (James *et al*., 1996).

### Plant transformation and growth

*Agrobacterium tumefaciens*‐mediated transformation of Arabidopsis was performed by floral dip (Clough and Bent, 1998) and transformation of *A. chinensis* var. *chinensis* ‘Hort16A’ was performed as previously described (Wang *et al*., 2007, 2010), using media supplemented with kanamycin (150 mg L^−1^) for the selection of transformants and timentin (300 mg L^−1^) to restrict the bacterial overgrowth. For transformation of fast‐flowering ‘Hort16A’ U6‐CEN4#7 (Varkonyi‐Gasic *et al*., 2019), the same method was adopted, but hygromycin (10 mg L^−1^) was used for the selection of transgenic lines. Cultures were maintained in a growth room with temperature at 24 ± 2 °C and 16‐h photoperiod with cool white fluorescent light (35–45 μmol m^−2^ s^−1^). Rooted transgenic plants were established in the growth room with controlled temperature and light conditions (temperature 22 °C, 16 h/8 h light/dark), then acclimatized in controlled glasshouse growth rooms (temperature min 18 °C/max 30 °C night/day, 14 h/10 h light/dark). Larger plants (>30 leaves) were transferred to the glasshouse and grown at ambient conditions. To evaluate budbreak frequencies, plants were defoliated in June and monitored for budbreak. Scoring was performed 4 weeks later. Twenty distal buds of each line were evaluated for growth stage and assigned a dormant, budbreak or shoot outgrowth status. Plants were photographed in August to capture a visible difference in appearance of evergrowing and control lines. Observations were carried out over three seasons (2019, 2020 and 2021).

### Genomic DNA extraction and genotyping

Genomic DNA was extracted from the young leaf tissue using DNeasy plant Mini Kit (Qiagen) following the manufacturer's instruction. PCR amplification was performed using iProof High‐Fidelity DNA Polymerase (Bio‐Rad) with gene or allele‐specific oligonucleotide primers. The PCR conditions were as follows: initial denaturation at 98 °C for 5 min, 35 cycles of denaturation at 98 °C for 15 s, annealing and extension at ≥58 °C for 15 s, final extension at 72 °C for 15–30 s/kb. The annealing temperature for allele‐specific amplification was 63 °C (allele I) and 65 °C (allele II). Amplification products were analysed by agarose gel electrophoresis, purified using the DNA Clean & Concentrate Kit (Zymo Research) and sent for sequence analysis (Macrogen, www.macrogen.com). For subsequent verification, amplification product were cloned into pJET1.2/blunt cloning vector supplied in the CloneJET PCR cloning kit (Thermo Fisher Scientific) and at least four clones of each line were subjected to sequence analysis. Data were analysed using the Geneious ClustalW. Oligonucleotide primer sequences are listed in Table S5.

### RNA‐seq

Total RNA of axillary buds of *Acbft* mutants and controls (harvested in May and July 2020) was extracted using the Spectrum Plant Total RNA kit (Sigma‐Aldrich, St. Louis, MO). Three biological replicates were used. Library construction, sequencing and bioinformatic analyses were performed at Novogene (www.en.novogene.com) (Illumina NovaSeq 6000, 150 bp paired‐end reads, 30 million reads per sample). Uniquely mapped reads to the *A. chinensis* Red5 genome (Pilkington *et al*., 2018) were interrogated for expression, which was presented as average fragments per kilobase of transcript per million reads (FPKM) ± SE of three biological replicates. Differentially expressed genes (DEGs) were identified using DESeq (Anders and Huber, 2010) by Novogene. For functional classification of DEGs, gene annotations were obtained with BLAST v.2.6.0 (Altschul *et al*., 1997) using *A. chinensis* Red5 amino acid sequences querying the Arabidopsis TAIR10_pep database, which were then used for Gene ontology (GO) analysis (Botstein *et al*., 2000) at the Gene Ontology Resource (http://geneontology.org/). Hierarchical clustering was performed using Morpheus (https://software.broadinstitute.org/morpheus/).

### Phylogeny

Sequence alignment of kiwifruit nucleic acid sequences was performed using Geneious MUSCLE alignment and the phylogenetic tree was built with Geneious tree builder, using the Neighbour‐Joining method, with 1000 bootstrap replicates.

### Yeast two‐hybrid assays

The assay was performed as described previously (Voogd *et al*., 2017). Briefly, bait and prey constructs were selected on minimal media lacking Leu or Trp (bait or prey respectively), followed by mating on YPAD plates and selection on minimal media lacking both Leu and Trp. The screening was performed on media lacking Trp, Leu and His and supplemented with 1, 3 and 5 mM 3‐amino‐1,2,4‐triazole (3AT). Plates were incubated for 3 days at 30 °C and scored for growth.

## Conflict of interest

The authors declare no conflict of interest.

## Authors' contributions

EV‐G and JP conceived the study, DH, CV and EV‐G designed the experiments, DH, CV and BY conducted the experiments. DH generated and maintained the kiwifruit transgenic lines, MM‐S generated the Arabidopsis transgenic lines. DH, CV, JP and EV‐G analysed the data. DH, EV‐G, JP and CV wrote the manuscript and ACA contributed to the final version of the manuscript. All authors approved the manuscript.

## Supporting information


**Figure S1** A phylogenetic tree of plant PEBP proteins.
**Figure S2** Kiwifruit protein interactions detected by yeast two‐hybrid assays.
**Figure S3** Mutagenesis of *AcBFT2*.
**Figure S4** Phenotyping of *Acbft* and control kiwifruit lines.
**Figure S5** Fruit and seed development after pollination with ‘Bruce’ pollen in a fast‐flowering *cen4* mutant and an *Acbft cen4* double mutant kiwifruit.
**Figure S6** GO‐term categories enriched in *Acbft* kiwifruit lines compared with controls.
**Figure S7** Expression of *AcFD* (Acc05237) in *Acbft* and control kiwifruit lines (top), axillary buds collected from wild‐type field‐grown plants at monthly intervals (middle) and axillary buds collected from excised canes exposed to cold over 4 weeks (bottom).

**Table S1** RNA‐seq analysis of *Acbft* and control kiwifruit lines collected in May and July, showing FPKM values of three biological replicates for each sample.
**Table S2** Differentially expressed genes between *Acbft* and control kiwifruit lines in May.
**Table S3** Differentially expressed genes between *Acbft* and control kiwifruit lines in July.
**Table S4** The common set of differentially expressed genes in May and July between *Acbft* and control kiwifruit lines.
**Table S5** Oligonucleotide primers used in this study.
