# Supplementary material for: CRISPR‐Cas9‐mediated mutagenesis of kiwifruit BFT genes results in an evergrowing but not early flowering phenotype
Source: Plant Biotechnol J. 2022 Jul 26;20(11):2064–76. doi: 10.1111/pbi.13888 (PMC9616528; doi:10.1111/pbi.13888)
Supplement: Supplementary file 1 — Figure S1 A phylogenetic tree of plant PEBP proteins. Figure S2 Kiwifruit protein interactions detected by yeast two‐hybrid assays. Figure S3 Mutagenesis of AcBFT2. Figure S4 Phenotyping of Acbft and control kiwifruit lines. Figure S5 Fruit and seed development after pollination with ‘Bruce’ pollen in a fast‐flowering cen4 mutant and an Acbft cen4 double mutant kiwifruit. Figure S6 GO‐term categories enriched in Acbft kiwifruit lines compared with controls. Figure S7 Expression of AcFD (Acc05237) in Acbft and control kiwifruit lines (top), axillary buds collected from wild‐type field‐grown plants at monthly intervals (middle) and axillary buds collected from excised canes exposed to cold over 4 weeks (bottom). [file PBI-20-2064-s001.docx]

Article title: CRISPR-Cas9-mediated mutagenesis of kiwifruit *AcBFT* genes results in an evergrowing, but not early flowering, phenotype

Authors: Dinum Herath, Charlotte Voogd, Matthew Mayo-Smith, Bo Yang, Andrew C Allan, Joanna Putterill, Erika Varkonyi-Gasic

Figure S1


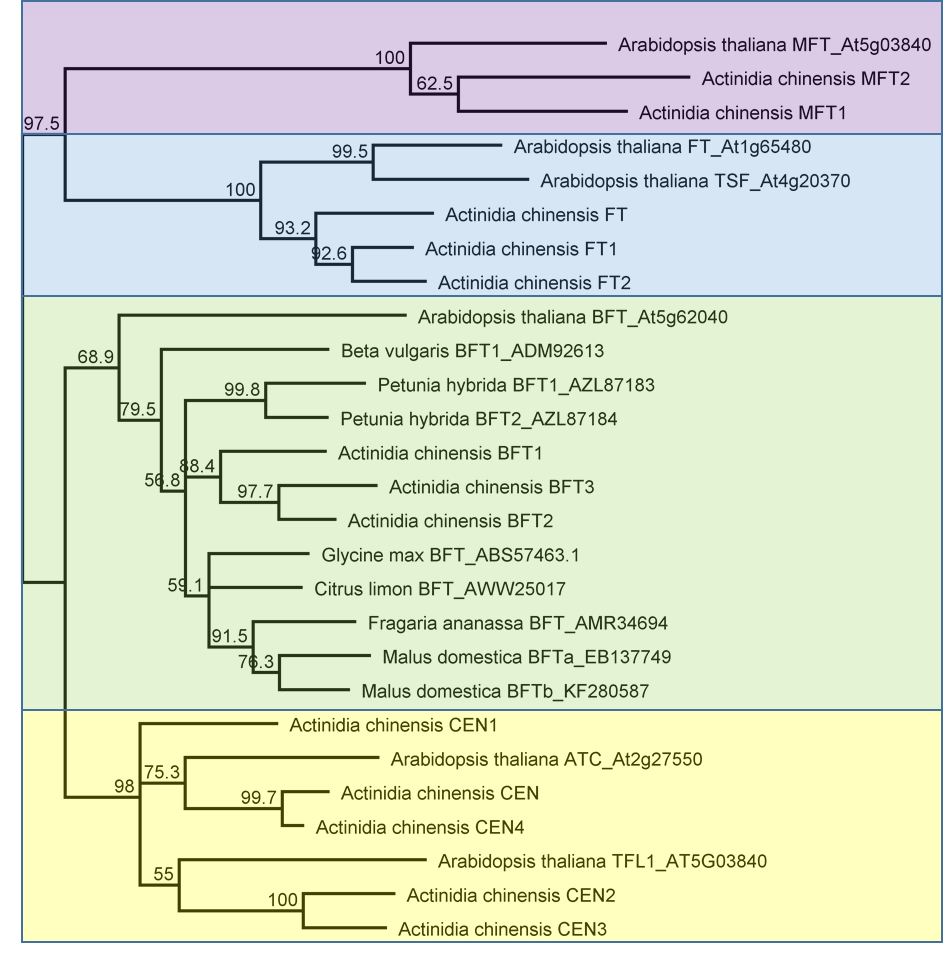


Figure S1. A phylogenetic tree of plant PEBP proteins. Kiwifruit *Actinidia chinensis* and Arabidopsis MFT, FT, and TFL1/CEN are highlighted purple, blue and yellow, respectively. BFT proteins from indicated plant species are highlighted green.

Figure S2


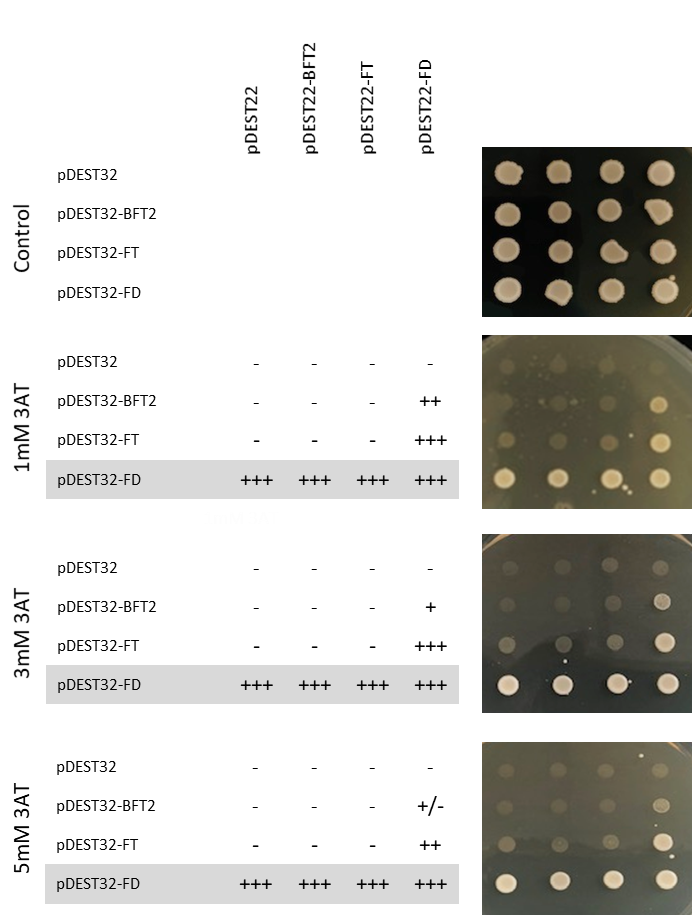


Figure S2. Kiwifruit protein interactions detected by yeast two-hybrid assays. +++, very strong interaction; ++, strong interaction; +, moderate interaction; +/−, weak interaction; −, no interaction. The rows show the pDEST32 bait vectors and the columns the pDEST22 prey vectors. Kiwifruit FD bait (pDEST32-FD, shaded grey) showed strong auto-activation and thus was excluded from the analysis. 3AT = 3-amino-1,2,4-triazole.

Figure S3


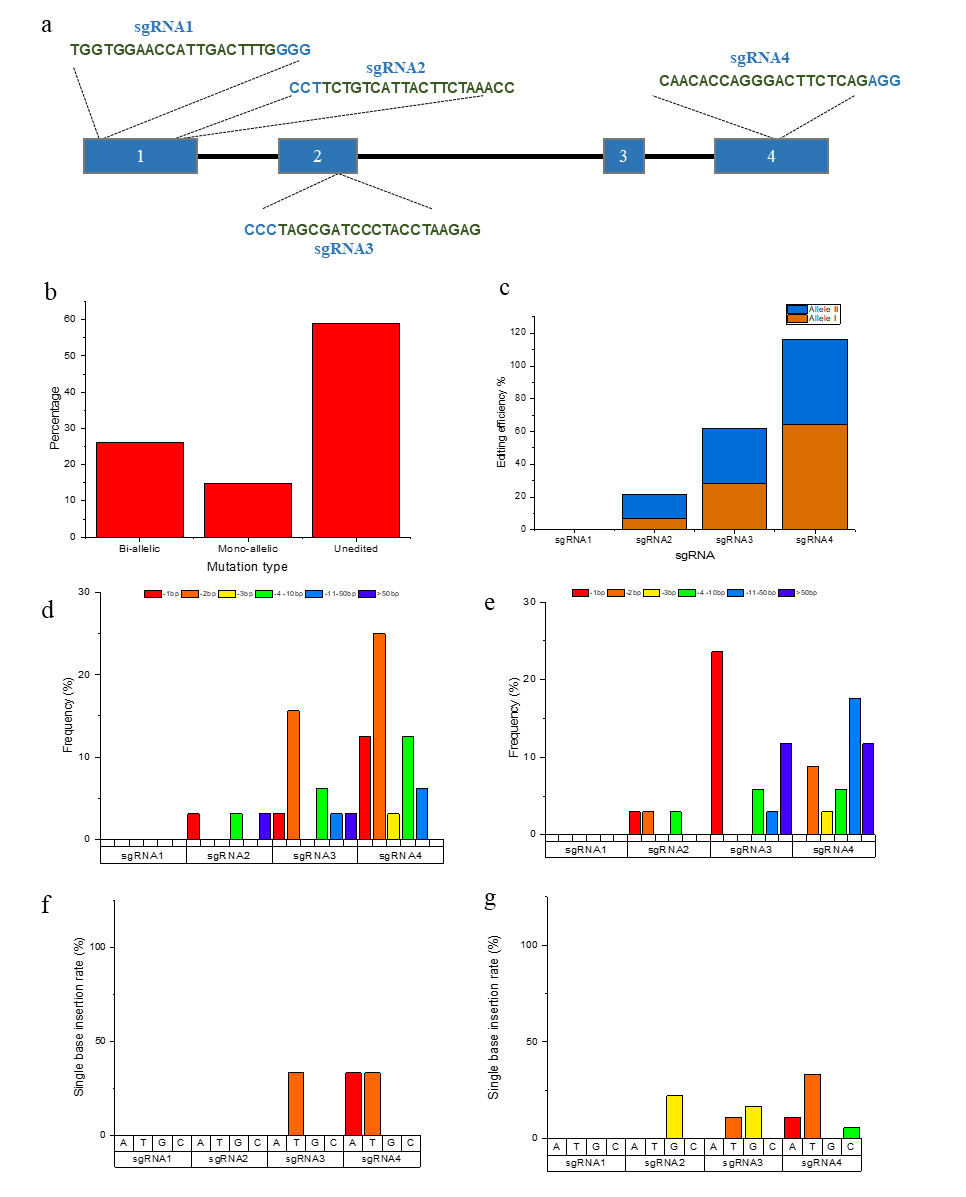


Figure S3. Mutagenesis of *AcBFT2*. (a) Schematic of *AcBFT2* gene and the positions of editing target sites (sgRNA1-sgRNA4); the blue boxes indicate exons and the PAM sites are highlighted in blue. (b) The frequency of bi-allelic and mono-allelic mutations (c) Editing efficiency of each sgRNA (d) Editing frequency in each target site in *AcBFT2* allele I (e) Editing frequency in each target site in *AcBFT2* allele II. (f) Single base insertion rate at each guide in *AcBFT2* allele I. (g) single base insertion rate at each guide in *AcBFT2* allele II.

Figure S4


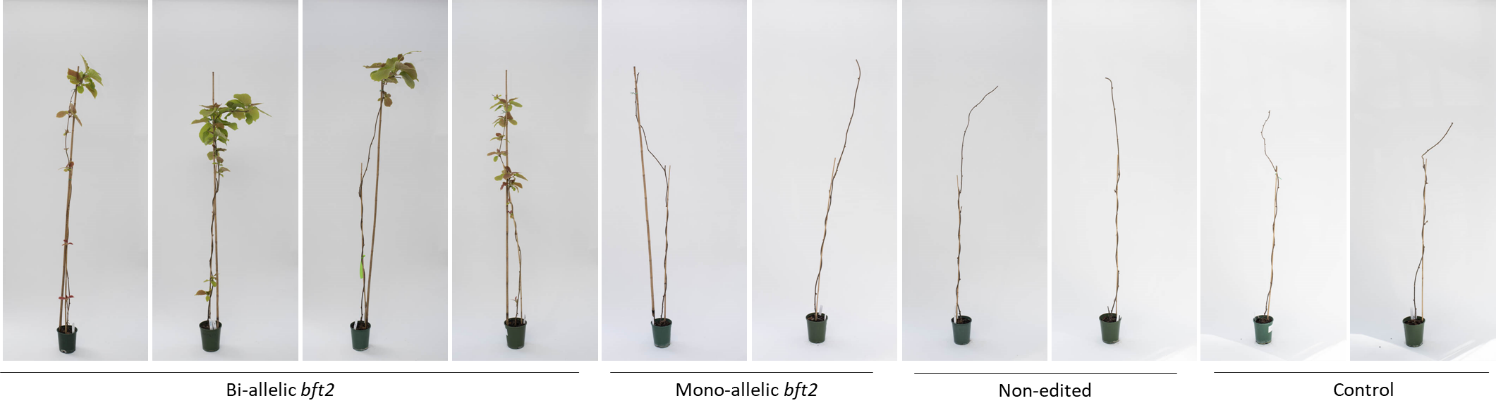


Figure S4. Phenotyping of *Acbft* and control kiwifruit lines. Representative *Acbft* lines with bi-allelic edits in *Acbft2* (left) showed earlier budbreak compared with the control lines (right). Transgenic lines with edits in one allele or no edits (middle) resembled the control lines. All plants were defoliated and maintained in the glasshouse without supplemented chilling. Photographs were taken in August 2019.

Figure S5


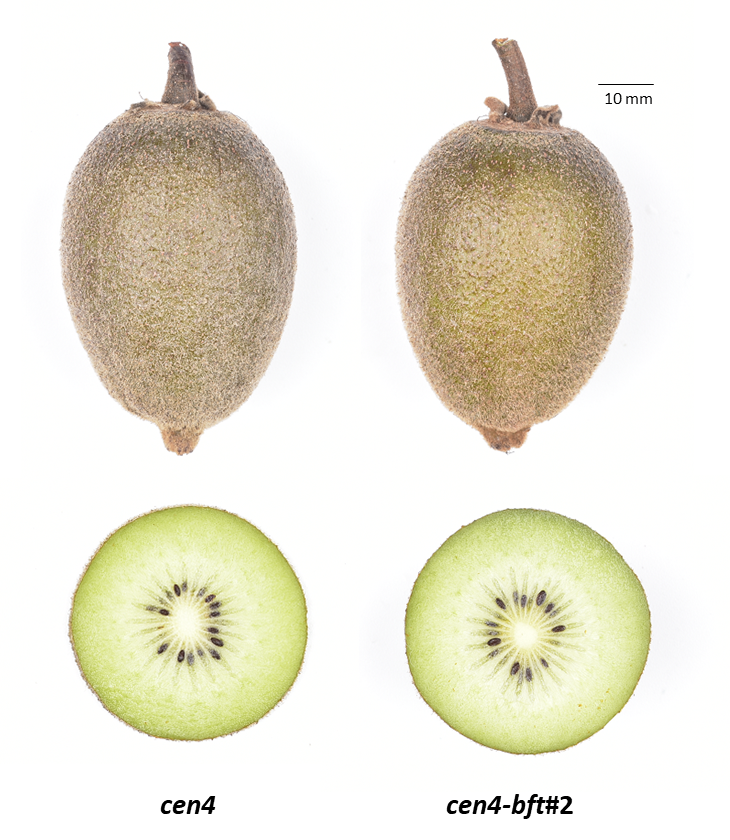


Figure S5. Fruit and seed development after pollination with ‘Bruce’ pollen in a fast-flowering *cen4* mutant and an *Acbft cen4* double mutant kiwifruit.

Figure S6


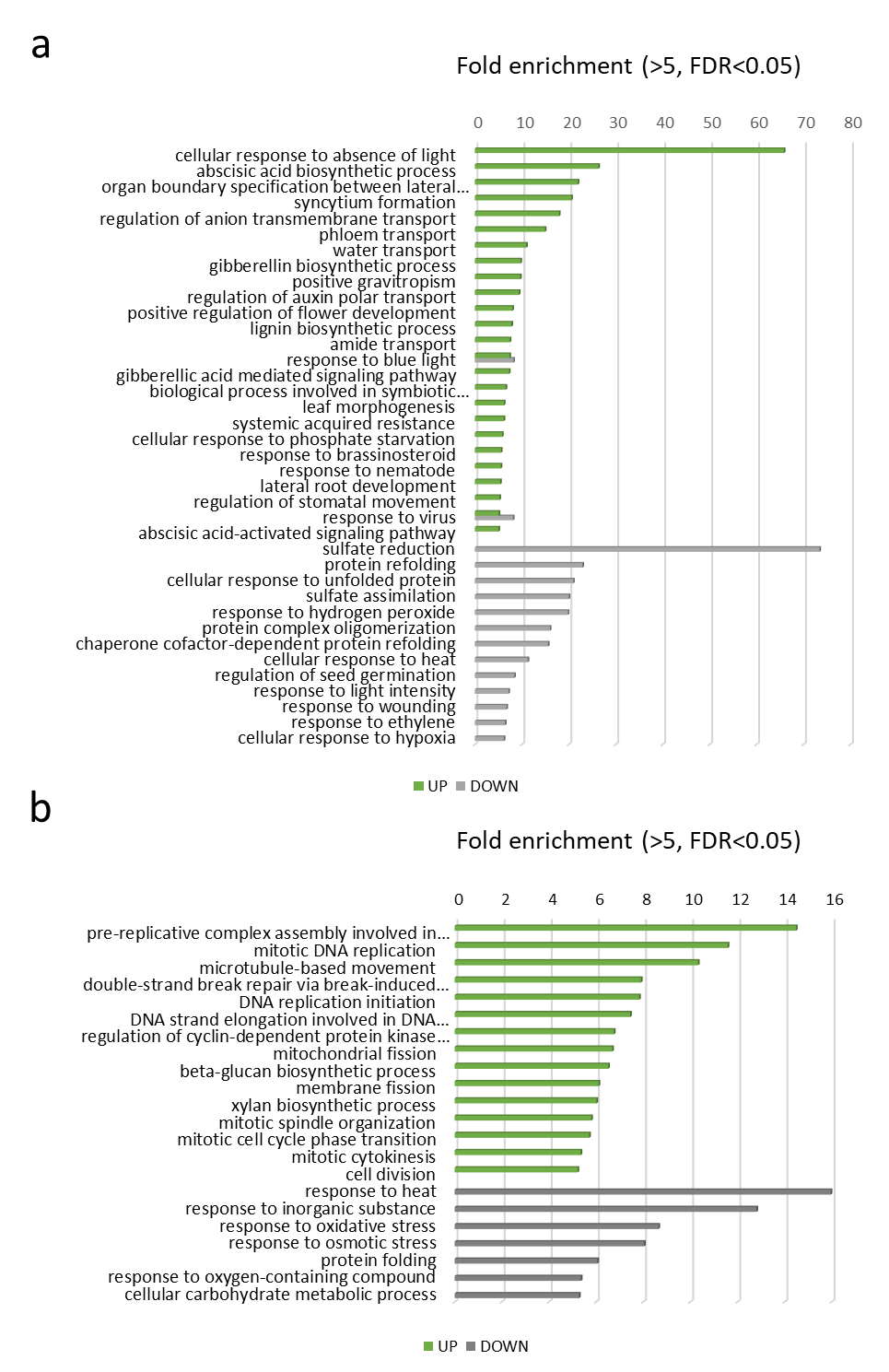


Figure S6. GO-term categories enriched in *Acbft* kiwifruit lines compared with controls. Enriched categories of DEGs in *Acbft* lines at May (a) and July (b). Only top categories with the minimum of 5-fold enrichment (FDR<0.05) are presented.

Figure S7


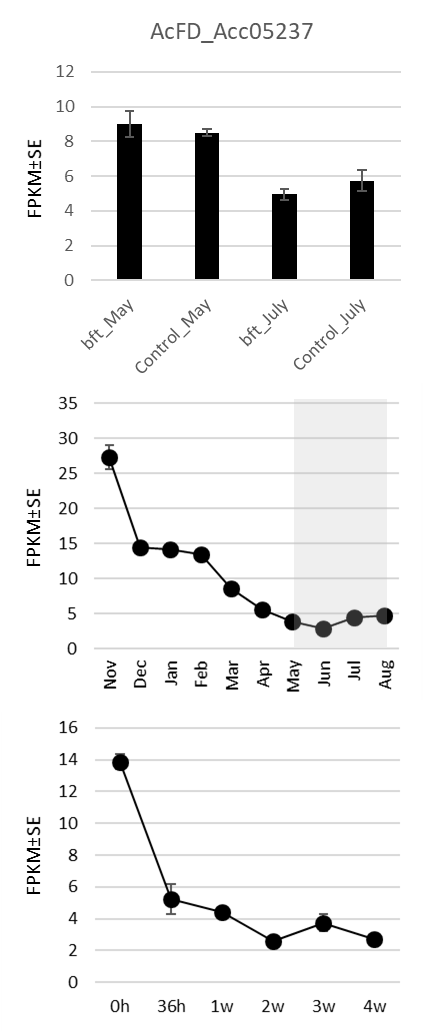


Figure S7. Expression of *AcFD* (Acc05237) in *Acbft* and control kiwifruit lines (top), axillary buds collected from wild-type field-grown plants at monthly intervals (middle) and axillary buds collected from excised canes exposed to cold over 4 weeks (bottom). The bars and circles represent mean FPKM±SE of three biological replicates. The period of no visible growth during winter in the field is shaded grey.
